# The iron-sulfur cluster assembly factor FDX2 is required for tumor initiation but not for growth of established tumors in transplantation models

**DOI:** 10.1016/j.jbc.2026.113200

**Published:** 2026-05-27

**Authors:** Eifumi Hashimoto, Mai Ohuchi, Miyuki Nomura, Shuko Miyahara, Kayoko Hayashi, Masatoshi Saito, Yoji Yamashita, Muneaki Shimada, Hidekazu Yamada, Nobuhiro Tanuma

**Affiliations:** 1 Division of Cancer Chemotherapy, Miyagi Cancer Center Research Institute, Natori, Japan; 2 Department of Biochemical Oncology, Tohoku University Graduate School of Medicine, Sendai, Japan; 3 Department of Obstetrics and Gynecology, Tohoku University Graduate School of Medicine, Sendai, Japan

**Keywords:** iron-sulfur cluster, iron-sulfur protein, oxygen, hypoxia, cancer biology, cancer metastasis, ovarian cancer, gene knockout, cellular senescence

## Abstract

Iron-sulfur (Fe-S) clusters bind to Fe-S proteins and are required for their function and/or structural stability. Recent work reveals an essential role for Fe-S cluster biosynthesis in cancer cell proliferation *in vitro*, but how Fe-S cluster metabolism contributes to tumor activity *in vivo* is unclear. Here we report analysis suggesting a stage-specific requirement for FDX2, a critical component of the Fe-S cluster assembly complex, in cancer progression. Using inducible loss-of-function transplant models of a human ovarian cancer line, we show that FDX2 is required for tumor initiation and metastasis but not for growth of established tumors in mice. We report global upregulation of Fe-S proteins under low oxygen conditions and concomitant attenuation of FDX2 loss-mediated disruption of many Fe-S proteins, enabling FDX2-independent proliferation. Our findings highlight a differential requirement of Fe-S cluster biosynthesis for tumor metastasis *versus* growth and low oxygen-mediated mitigation of Fe-S protein loss promoted by FDX2 deficiency.

Iron–sulfur (Fe-S) clusters are ancient, ubiquitous cofactors that play indispensable roles in fundamental cellular processes. Clusters exist as 2Fe–2S, 4Fe–4S, or other forms, which are typically coordinated by Cys or His residues found in specific Fe-S proteins (1, 2). Among these proteins, Fe-S clusters participate directly in electron transfer reactions and diverse enzymatic processes and contribute to structural stability (1, 2). Consequently, Fe-S clusters are essential for mitochondrial respiration, DNA replication and repair, and other cellular functions (3, 4, 5, 6, 7, 8). Proper biosynthesis and maintenance of Fe-S proteins are therefore critical for cell viability, and their dysfunction is linked to many human diseases (1, 2, 9, 10).

Cancer cells proliferate within hypoxic microenvironments, and their ability to adapt to low oxygen availability is a key determinant of tumor progression (11). Relevant to the relationship between oxygen levels and Fe-S clusters, previous studies have implicated Fe-S clusters and Fe-S proteins in cell growth and survival under high oxygen conditions (12, 13). For example, Baik *et al.* demonstrated that hyperoxia (50% O_2_) induces global degradation of Fe-S proteins (13). NFS1, a component of the core Fe-S cluster assembly complex, reportedly exerts anti-ferroptotic activity by limiting iron-starvation responses and blocking accumulation of cellular free iron (Fe^2+^), an important function under high oxygen environments, including standard cell culture conditions (∼20% O_2_) (12). Accordingly, Fe-S cluster biosynthesis is generally considered essential for cell proliferation under standard culture conditions, which are hyperoxic relative to *in vivo* conditions (14, 15, 16, 17, 18). Nevertheless, little is known about how homeostasis of Fe-S clusters and Fe-S proteins is regulated under low oxygen conditions and how these activities impact cancer cell activity, even though Fe-S clusters are known to be highly susceptible to oxidative stress (1, 2). Frataxin (FXN), a core component of the mitochondrial Fe-S cluster assembly machinery, is required for efficient Fe-S cluster biosynthesis. Previous work has shown that hypoxia mitigates the downregulation of several Fe-S proteins in FXN-mutant cells, thereby attenuating associated proliferation defects (19).

Mitochondrial ferredoxin 2 (FDX2) is also a component of the Fe-S cluster biosynthesis machinery and acts as an electron donor within the core Fe-S cluster assembly complex (20, 21, 22). Recently, we and others showed that FDX2 is important for maintaining proper Fe-S protein levels and proliferation in several human cell lines (23, 24, 25). Specifically, in normal culture conditions, conditional FDX2 depletion in JHOC5 ovarian cancer cells promoted global Fe-S protein down-regulation and either senescence-like growth arrest or apoptosis, depending on TP53 status (25). However, FDX2 function in tumor biology, particularly *in vivo*, remains uncharacterized as JHOC5 cells are not tumorigenic in mice. Moreover, it remains unclear whether oxygen tension modulates cellular dependency on FDX2 and Fe-S cluster biosynthesis.

To address these questions, here we developed a new ovarian cancer line with inducible FDX2 knockout to test FDX2 function in tumorigenesis *in vivo* and then asked whether FDX2 is required for tumor formation *versus* growth in transplantation models. Using multiple *in vivo* models, we report a stage-specific requirement for Fe-S cluster biosynthesis and reveal a previously underappreciated link between hypoxia and Fe-S protein homeostasis.

## Results

### FDX2 is required to prevent senescence-like proliferation arrest under ambient oxygen conditions

**Figure 1:**
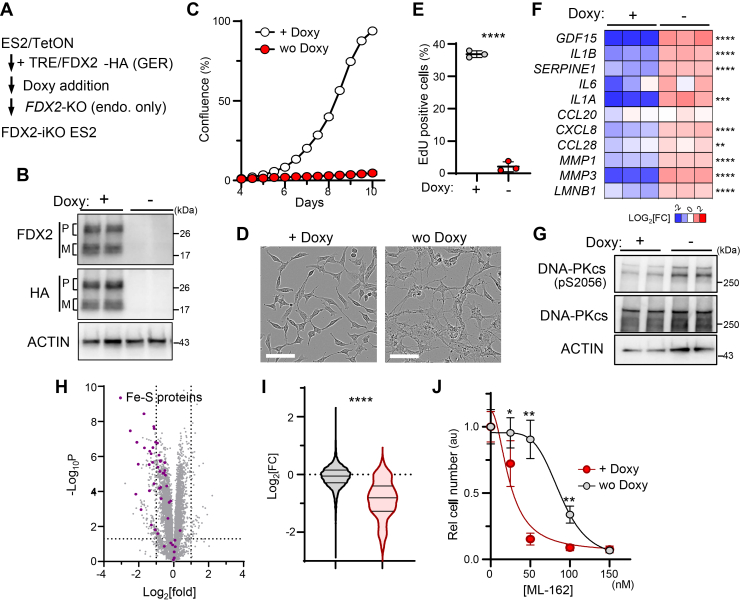
**FDX2-deficiency induces senescence-like proliferation arrest in ES2 cells under ambient oxygen.***A*, schematic showing generation of FDX2-iKO ES2 cells. Cells were first engineered to express exogenous C-terminally HA-tagged FDX2 (FDX2-HA) in a Doxycyclin (Doxy)-dependent manner. GER denotes Genome-editing resistant (see Fig. S1*A* for details). Then, in the presence of Doxy, the endogenous *FDX2* was knocked out by genome editing, such that Doxy withdrawal promoted FDX2 loss. Genome-edited cells were maintained in Doxy-containing medium. *B*, Western blot of FDX2-iKO ES2 cells before and after Doxy withdrawal. P and M denote premature (containing the mitochondrial presequence) and mature forms of FDX2, respectively. *C*, proliferation rate of FDX2-iKO ES2 cells cultured with or without Doxy. After a 4-days preculture with or without Doxy, cells were re-seeded into new culture plates to perform a real-time proliferation assay. Shown are averages of 3 biological replicates. *D*, representative phase contrast images of FDX2-iKO ES2 cells cultured 8 days with or without Doxy. Scale bars, 100 μm. *E*, the percentage of EdU-positive FDX2-iKO ES2 cells. Cells were cultured 6 days with or without Doxy and then pulse-labeled with EdU for 30 min n = 3 biological replicates. *F*, expression of mRNA encoding indicated SASP factors in FDX2-iKO ES2 cells. Cells were cultured 6 days in the presence or absence of Doxy before RNA-seq analysis. n = 3 biological replicates. *G*, levels of Ser2056-phosphorylated (pS2056) or total DNA-PKcs in FDX2-iKO ES2 cells 6 days after FDX2-KO induction. *H*, Volcano plot showing effects of FDX2 loss on the ES2 cell proteome. Cells were cultured 6 days with or without Doxy prior to MS-based proteome analysis. Fe-S proteins are highlighted in red. n = 4 biological replicates. *p* values were determined by two-tailed *t* test. *I*, analysis of results shown in H to show effects of FDX2 knockout on all proteins *versus* Fe-S proteins only. *J*, enhanced sensitivity of FDX2-deficient cells to the GPX4 inhibitor ML-162. Cells were cultured 5 days with or without Doxy and then treated 24 h with ML-162 at indicated concentrations. n = 6 biological replicates. Data are presented as means plus SD (*C*, *E*, *J*). ∗*p* < 0.05, ∗∗*p* < 0.01, ∗∗∗∗*p* < 0.0001 as determined by two-tailed *t* test (*E*, *F*, *I*) and by two-tailed Mann–Whitney *U* test (*J*).

We first generated ES2 human ovarian cancer cells expressing exogenous HA-tagged FDX2 under control of the TetON system and then knocked out the endogenous *FDX2* gene in these cells by CRISPR-Cas9 genome editing (Fig. 1*A*). The FDX2-HA construct in these cells harbored silent mutations in the target site to avoid recognition by the genome editing machinery (Fig. S1*A*). To avoid growth inhibition caused by FDX2 loss, genome-edited cells were cultured in the presence of doxycycline (Doxy). Cells were screened by Western blotting, and we obtained 2 knockout clones (hereafter referred to as FDX2-inducible KO (-iKO) clones) showing inducible FDX2 deletion in the absence of Doxy in the medium (Fig. 1*B*). Unless specified, we routinely cultured FDX2-iKO cells for 4 days in the presence or absence of Doxy to induce FDX2 loss prior to analysis.

Under normal culture conditions, FDX2 loss promoted robust proliferation arrest (Fig. 1*C*). FDX2-deficient cells exhibited an enlarged, flattened morphology (Fig. 1*D*) and strongly suppressed DNA synthesis, based on an EdU-incorporation assay (Fig. 1*E*). RNA-seq followed by gene-set enrichment analysis revealed significant downregulation of genes related to cell-cycle progression after FDX2 depletion (Fig. S1*B*). We also observed upregulation of transcripts encoding major senescence-associated secretory phenotype (SASP) factors (26, 27) (Fig. 1*F*). Western blot analysis revealed increased levels of phosphorylated DNA-PKcs after FDX2 loss, suggestive of DNA damage (Fig. 1*G*). We did not observe significant death of FDX2-deficient cells up to day 10. Overall, we conclude that FDX2 deficiency induces senescence-like proliferation arrest in ES2 cells, although we did not detect SA-β-gal activity (data not shown) or activation of CHK1 and CHK2 (28) (Fig. S1*C*).

We next performed proteomic analysis of FDX2-depleted *versus* WT cells. That analysis revealed downregulated levels of many Fe-S proteins after FDX2 loss (Figs. 1, *H*, *I* and S1*D*), an outcome confirmed by a capillary-based immunoassay (Fig. S1*E*). Among them, those related to DNA repair or replication tended to decline more prominently following FDX2 depletion (Fig. S1*F*), as previously observed in JHOC5 cells (25). We observed no clear relationship between changes in Fe–S protein levels and corresponding mRNA levels (Fig. S1*G*). Next, we asked whether FDX2 loss sensitizes ES2 cells to ferroptosis, as we previously reported in JHOC5 cells (24). To this end, we first assessed vulnerability of FDX2-iKO ES2 cells to treatment with two ferroptosis inducers: the glutathione peroxidase 4 (GPX4) inhibitor ML-162 and the ferroptosis suppressor protein 1 (FSP1) inhibitor iFSP1 (29) (Fig. S1*H*). Under FDX2-proficient conditions (+Doxy), ML-162 treatment induced significant cell death, although iFSP1 treatment had minimal effects, even when combined with ML-162 (Fig. S1*I*). Therefore, we performed comparable experiments with ML-162 alone, in the presence or absence of Doxy, and observed sensitization of FDX2-deficient cells to ML-162-induced cell death (Fig. 1*J*). These results suggest that FDX2 deficiency in ES2 cells under normal culture conditions down-regulates Fe-S protein levels and increases cell vulnerability to ferroptosis, consistent with our previous observations in JHOC5 cells (25).

### FDX2 is required for tumor initiation and metastasis

**Figure 2:**
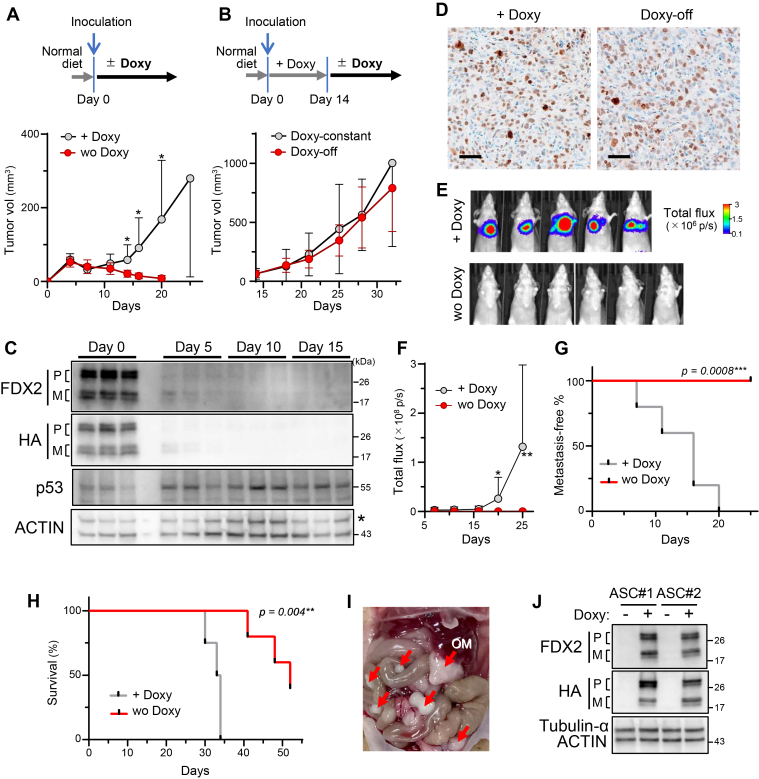
**FDX2 is required for tumor formation and metastasis but not growth of established tumors in xenograft models.***A*, volumes of xenograft tumors derived from FDX2-iKO ES2 cells. Nude mice were inoculated *sc* with FDX2-iKO cells (grown *in vitro* with Doxy) on day 0 and then fed a diet with or without Doxy. The number of tumors analyzed in with- and without-Doxy groups was n = 8 and n = 10, respectively. *B*, effects of Doxy withdrawal on growth of established tumors. Mice inoculated *sc* with FDX2-iKO cells were maintained on a Doxy-containing diet for 14 days and then divided into 2 groups: one that remained on the Doxy-containing diet (Doxy-constant) and the other switched to a Doxy-free diet (Doxy-off). The number of tumors analyzed in the with- and without-Doxy groups was n = 7 and n = 10, respectively. *C*, WB analysis of FDX2-iKO tumors from mice subjected to Doxy withdrawal, as described in B. Tumor samples were collected 0, 5, 10, or 15 days after switching mice from the Doxy-containing to Doxy-free diet and blotted for FDX2. Samples were also blotted with a human p53-specific antibody that does not react with mouse p53, confirming that individual tumors exhibit comparable proportions of FDX2-iKO cells. ACTIN served as loading control; asterisk indicates unrelated cross-reacting band. *D*, IHC analysis of Ki-67 in FDX2-iKO ES2 tumors before (+Doxy) and after Doxy removal (Doxy-off). Tumor-bearing mice maintained on the Doxy-containing diet were switched to the Doxy-free diet 10 days prior to sample collection. Scale bars, 50 μm. *E*, formation of metastases from FDX2-iKO ES2 cells in an *iv* transplantation model. Mice inoculated iv *via* the tail vein with FDX2-iKO cells were divided into 2 groups on day 0 as in A. Tumor burden was monitored by luciferase-based imaging over a 3-week period. Shown are representative bioluminescent images at day 25. n = 5 and 6 mice for + Doxy and wo Doxy groups, respectively. *F*, qQuantification of metastatic burden over time (day 5–25) by bioluminescent imaging, as shown in E. *G*, metastasis-free survival of mice inoculated iv with FDX2-iKO cells as described in *E*. *H*, survival of mice transplanted *ip* with a low number (3 × 10^5^) of FDX2-iKO cells. Mice inoculated were divided into 2 groups as in A: n = 4 and 5 mice for + Doxy and -Doxy groups, respectively. (*I*) Representative view of peritoneal dissemination of FDX2-iKO ES2 cells. Arrows indicate major tumor nodules. OM, omentum. (*J*) WB analysis of tumor cells isolated from ascites fluid of mice in the -Doxy group. Cells were further cultured for 3 days under 3% O2 conditions, with or without Doxy. Data are presented as mean plus SD (*A*, *B*, *F*). ∗*p* < 0.05, ∗∗*p* < 0.01, ∗∗∗∗*p* < 0.0001 as determined by two-tailed *t* test (*A*, *B*), the Mann–Whitney *U* test (*F*), or log-rank test (*G*, *H*).

To assess FDX2 function in tumor growth *in vivo*, we first examined the effects of FDX2 deficiency in a subcutaneous (*sc*) tumor model. We transplanted FDX2-iKO ES2 cells that had been maintained in Doxy *in vitro* into the dorsal flank of nude mice and then immediately divided mice into 2 groups, one fed a Doxy-containing diet and the other a Doxy-free diet (Fig. 2*A*, upper). In mice fed the Doxy-containing diet, tumors formed and continued to grow (Fig. 2*A*, lower), while tumor formation was significantly suppressed in mice fed the Doxy-free diet. Specifically, in the Doxy-free group, immediately after transplantation FDX2-iKO cells formed small nodules, but these nodules did not grow and instead regressed and were resorbed within 2 to 3 weeks (Fig. 2*A*, lower), indicating that FDX2 is required for tumor initiation in this model.

We next asked whether FDX2-deficiency would alter the growth of established tumors. To do so, we transplanted nude mice with FDX2-iKO cells, fed mice a Doxy-containing diet for 14 days to allow tumor engraftment, and then divided those mice into 2 groups (Fig. 2*B*, upper): one that continued the Doxy-containing diet for the rest of the experimental period (Doxy-constant) and the other switched to a Doxy-free diet (Doxy-off). We observed no significant differences in tumor growth between these 2 groups (Fig. 2*B*, lower). Western blot analysis of tumor tissue showed a rapid decline in FDX2 protein levels within 5 days of switching mice from a + Doxy to -Doxy diet, and FDX2 protein was no longer detectable in these tissues 10 days after Doxy-removal (Figs. 2*C* and S2*A*). Moreover, 10 days after Doxy removal, the Ki-67-index of tumors was comparable to that seen in tumors from mice maintained on the + Doxy diet, although the index was low in severely hypoxic regions in both groups, based on CA9 staining (Figs. 2*D* and S2, *B* and *C*). These results show that in a *sc* transplantation model, FDX2 is required for tumor initiation but not growth of established ES2-derived tumors.

Given evidence obtained from the *sc* model that FDX2 is required for tumor engraftment, we examined tumor establishment following intravenous (*iv*) injection, as engraftment capacity of tumor cells is thought to be associated with their ability to colonize distant sites (30, 31). To do so, we injected nude mice *iv* with FDX2-iKO cells (harboring a firefly luciferase gene) and maintained them on either a Doxy-containing or -Doxy-free diet. Bioluminescence imaging (conducted 1–4 weeks post-transplantation) revealed tumor outgrowth primarily in the lungs of + Doxy mice (Fig. 2*E*), while the –Doxy group showed a markedly suppressed tumor burden (Fig. 2, *E*–*G*).

We next evaluated the effects of FDX2 deletion in an intraperitoneal (*ip*) transplantation model. In mice inoculated *ip* with 3 × 10^5^ cells, the -Doxy group showed delayed tumor burden (Fig. S3*A*) and prolonged overall survival relative to the + Doxy group (Fig. 2*H*). In both groups, transplanted cells formed multiple metastatic nodules, predominantly in the omentum (Fig. 2*I*). Immunohistochemical analysis of omental metastatic lesions confirmed loss of FDX2 expression in the –Doxy tumors, whereas the Ki-67 index was comparable between groups (Fig. S3, *A* and *B*). Tumor cells isolated from ascites in -Doxy mice indeed lacked FDX2 expression, which was restored upon Doxy exposure *ex vivo* (Fig. 2*J*). These results indicate that FDX2 depletion suppresses peritoneal dissemination of ES2 cells, although this effect was less pronounced than that seen during tumor initiation in the *sc* model (Fig. 2*A*). The findings also suggest that FDX2 dependency may decrease after metastatic outgrowth, similar to that observed during the maintenance phase (Fig. 2*B*) in the *sc* model. Accordingly, we observed no significant differences in survival between the -Doxy and +Doxy groups when 1 × 10ˆ6 cells were inoculated (Fig. S3*C*).

### Low oxygen rescues FDX2 deficiency in ovarian cancer cells

**Figure 3:**
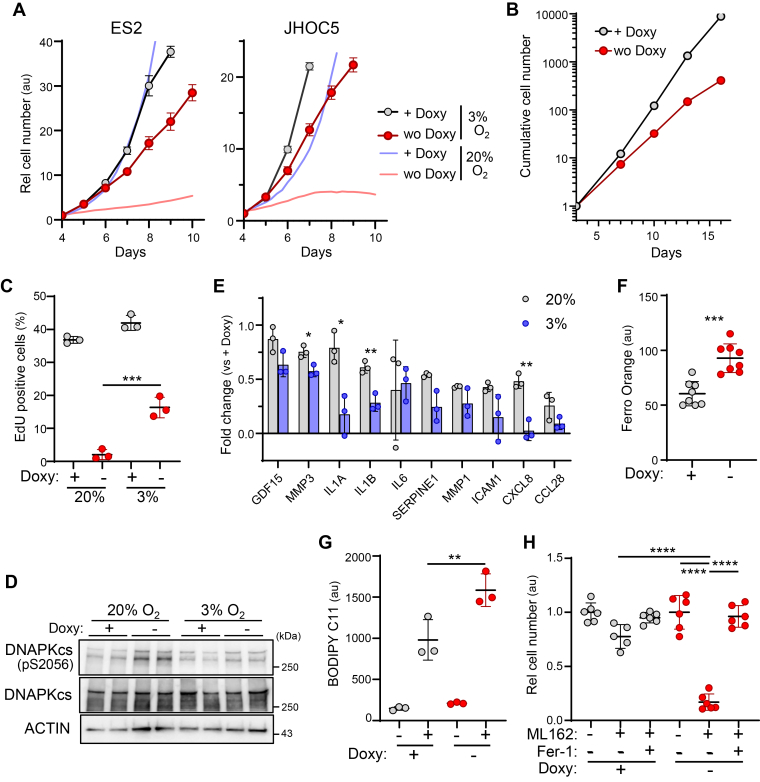
**Low oxygen rescues FDX2 loss-induced senescence phenotypes in ovarian cancer cells.***A*, growth of FDX2-iKO ES2 and FDX2-iKO JHOC5 cells cultured with or without Doxy at 3% O_2_. After a 4-days preculture with or without Doxy at 20% O_2_, cells were reseeded into new plates and cultured at 3% O_2_. Data represent means of 3 biological replicates. Growth curves at 20% O_2_ (representative curves are shown in Figure 1*C* for ES2 or reported elsewhere for JHOC5 (ref)) are also included. *B*, long-term proliferation assay of FDX2-iKO ES2 cultured with or without Doxy at 3% O_2_. After a 3-days preculture with or without Doxy at 20% O_2_, cells were reseeded into new dishes, cultured at 3% O_2_, and passaged at 3 to 4 days intervals. Cell numbers were determined at each passage. Data represent means of 3 biological replicates. *C*, the percentage of EdU-positive FDX2-iKO ES2 cells after pulse-labeling. After a 4-days preculture as in *A*, cells were cultured additional 3 days at indicated O_2_ concentrations and then pulse-labeled 30 min with EdU. Note that the percentages at 20% O_2_ are same as in Figure 1*E*. n = 3 biological replicates. *D*, levels of Ser2056-phosphorylated (pS2056) or total DNA-PKcs in FDX2-iKO ES2 cells cultured as in C. Note that the blots at 20% O_2_ are the same as in Figure 1*G*. *E*, comparison of SASP gene expression induced by FDX2 loss at 3% and 20% O_2_. RNA-seq data were analyzed for levels of transcripts encoding SASP factors either 2 (20% O_2_) or 3 (3% O_2_) days after 4-days preculture. Shown are relative expression levels compared to cells cultured with Doxy under each O_2_ condition. n = 3 biological replicates. *F*, cellular free Fe^2+^ levels in FDX2-iKO cells cultured with or without Doxy at 3% O_2_. n = 8 biological replicates. *G*, analysis of lipid peroxidation in FDX2-iKO ES2 cells. After a 4-days preculture with or without Doxy, cells were shifted to 3% O_2_, cultured another 2 days, and then treated 2 h with ML-162 or left untreated and incubated with BODIPY C11 probe. Fluorescent signals of oxidized BODIPY C11 were detected by flowcytometry. Shown are results of 3 independent experiments. *H*, sensitivity of FDX2-iKO ES2 cells to GPX4 inhibition under low oxygen conditions. Cells cultured 2 days at 3% O_2_ after a 4-day preculture as in G were treated for an additional day at 3% O_2_ with 100 nM ML-162 in the presence or absence of 10 μM Ferrostatin-1 (Fer-1). The number of viable cells was determined by SRB staining. n = 7 to 8 biological replicates for each group. Data are presented as means plus SD (*A*-*C*, *E*-*H*). ∗*p* < 0.05, ∗∗*p* < 0.01, ∗∗∗∗*p* < 0.0001 as determined by two-tailed *t* test (*F*, *E*) or by one-way ANOVA followed by a Tukey’s *post hoc* test (*C*, *G*, *H*).

The transplantation analysis described above suggests that under certain conditions FDX2 is dispensable for cell proliferation. Others have reported that hyperoxia (50% O_2_) induces active degradation and downregulation of many Fe-S proteins (13). Conversely, oxygen concentrations in the range of 1 to 3% O_2_ are commonly used to model tumor hypoxia *in vitro*, although such levels may fall within the range of physiologically relevant oxygen levels in certain tissues (*e.g.*, Refs. (32, 33, 34)). Previous work has shown that hypoxia attenuates proliferation defects of FXN-mutant cells (19). Accordingly, we hypothesized that lower *in vivo* oxygen levels relative to those seen *in vitro* may compensate for FDX2 loss. To test this hypothesis, we cultured FDX2-iKO ES2 cells in 3% O_2_, with or without Doxy, and found that the FDX2-deficient cells continued to proliferate under these conditions, although at a lower rate than did FDX2-proficient cells, in contrast to what we observed at 20% O_2_ (Fig. 3*A*, left). Transcriptome analysis confirmed upregulation of a HIF-1–driven gene expression program under 3% O2, both in the presence and absence of Doxy (Fig. S4*A*). Some HIF-1 target genes, including SLC2A3 and VEGFA, were upregulated in FDX2-deficient cells, even at 20% O_2_ (Fig. S4*A*). We obtained similar results in FDX2-iKO JHOC5 cells, suggesting that the rescue effect of low oxygen on FDX2 deficiency may be a general phenomenon (Fig. 3*A*, right). We also observed proliferation of FDX2-deficient ES2 cells at 1% O2 (Fig. S4*B*). In long-term proliferation assays, FDX2-deficient ES2 cells continued to proliferate steadily for more than 2 weeks (Fig. 3*B*). EdU-labeling analysis confirmed significantly increased DNA synthesis in FDX2-deficient cells grown in 3% compared to 20% O_2_ (Fig. 3*C*). Induction of DNA-PKcs phosphorylation and SASP factor gene expression by FDX2 loss was also decreased in cells grown at 3% compared with 20% O_2_ (Figs. 3, *C*–*E* and S4*C*). Overall, these results indicate that low oxygen conditions block induction of the senescence program by FDX2 loss.

We also asked whether ferroptosis-related phenotypes promoted by FDX2 loss, which were seen at 20% O_2_ (Fig. 1*J*), would be altered under low oxygen conditions. To address this question, we first compared free Fe^2+^ levels in cells grown at 3% O_2_ in the presence or absence of Doxy. We observed higher levels of cellular free Fe^2+^ in FDX2-deficient cells, likely due to impaired Fe-S cluster synthesis (35) (Fig. 3*F*). Furthermore, we observed a similar predisposition to ferroptosis in FDX2-iKO ES2 cells even under hypoxia, based on their increased sensitivity to ML-162, as evidenced by increased levels of lipid peroxidation and cell death, phenotypes that were completely blocked by treating cells with the ferroptosis inhibitor Fer-1 (Figs. 3, *G*, *H* and S4*D*). Given these results, we hypothesized that increases in ferroptosis may underlie reduced proliferation of FDX2-deficient relative to -proficient cells at 3% O_2_. However, Fer-1 did not affect the proliferation of FDX2-deficient ES2 cells at 3% O_2_ (Fig. S4*E*). These findings suggest that ferroptosis does not underlie the proliferation decreases seen in FDX2-deficient cells under low-oxygen conditions.

### Low oxygen mitigates Fe-S protein loss induced by FDX2 deficiency

**Figure 4:**
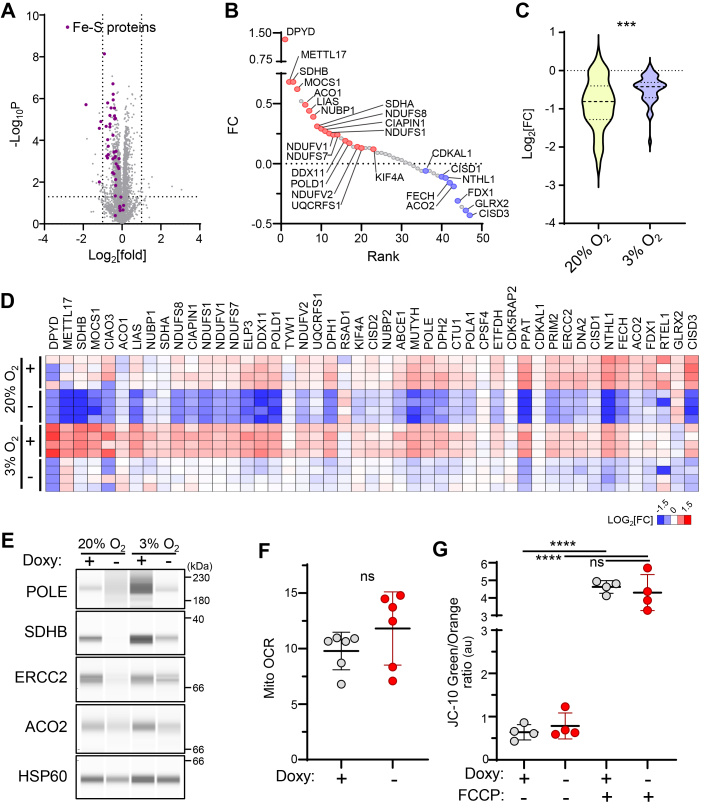
**Low oxygen antagonizes downregulation of Fe-S proteins and restores ETC functions impaired by FDX2 loss.***A*, Volcano plot showing effects of FDX2-KO on the ES2 cell proteome at 3% O_2_. After a 4-days preculture with or without Doxy, cells were cultured 3 more days at 3% O_2_, followed by MS-based proteome analysis. Fe-S proteins are highlighted in red. n = 4 biological replicates. *p* values were determined by two-tailed *t* test. *B*, Rank plot of all Fe-S proteins detected in proteome analysis. Fe-S proteins were ranked based on the extent of change in their levels seen in ES2 cells grown in the presence of Doxy at 3% compared to 20% O_2_. Shown are re-analyses of data in A and Figure 1*H*. Fe-S proteins showing statistically significant changes (two-tailed *t* test, *p* < 0.05) are highlighted in *red* or *blue*. *C*, comparison of effects of FDX2 deficiency on Fe-S protein levels in 20% and 3% O_2_ conditions. Note that the data at 20% O_2_ are also included in Figure 1*I*. *D*, heatmap showing relative levels of Fe-S proteins based on proteome analysis. Data are ordered according to the rank in B. n = 4 biological replicates. *E*, effects of FDX2 deficiency on levels of representative Fe-S proteins in FDX2-iKO ES2 cells grown in 20% and 3% O_2_ conditions, as determined by immunoassays. HSP60 served as loading control. *F*, mitochondrial oxygen consumption rate (OCR) in FDX2-iKO ES2 cells. After a 4-days preculture at 20% O_2_, with or without Doxy, cells were then cultured 3 more days in 3% O_2_, with or without Doxy, prior to Seahorse analysis. Rotenone/antimycin A-sensitive OCR was interpreted to represent mitochondrial OCR, as described in Methods. n = 6 biological replicates. *G*, analysis of mitochondrial depolarization using a JC-10 probe in FDX2-iKO ES2 cells grown in 3% O_2_. After a 4-days preculture with or without Doxy, cells were cultured 3 more days in 3% O_2_, with or without Doxy. Depolarization was induced by treatment with FCCP (added 30 min before probe loading). n = 4 biological replicates. Data are presented as means plus SD (F, G). ∗*p* < 0.05, ∗∗*p* < 0.01, ∗∗∗∗*p* < 0.0001 as determined by two-tailed *t* test (*C*, *F*) or by one-way ANOVA followed by a Tukey’s *post hoc* test (*G*).

To define mechanisms underlying proliferation of FDX2-KO cells under low oxygen conditions, we performed proteome analysis to compare levels of Fe-S proteins in FDX2 knockout cells grown at 3% and 20% O_2_. FDX2 loss induced global downregulation of Fe-S proteins both 3% O_2_ (Figs. 4*A* and S5*A*), as we previously observed at 20% O_2_. (Fig. 1*I*). However, in + Doxy conditions, steady-state levels of many known Fe-S proteins were significantly higher at 3% compared to 20% O_2_ (Fig. 4*B*), whereas their mRNA levels were comparable in both conditions (Fig. S5*B*). The magnitude of Fe-S protein downregulation mediated by FDX2 loss was also significantly greater at 20% compared to 3% O_2_ (Fig. 4*C*). Notably, levels of many Fe-S proteins, including METTL17, SDHB, MOCS1, and NDUFS1, in FDX2-KO cells grown at 3% O_2_ were comparable to those seen in FDX2-proficient cells at 20% O_2_, although levels of these proteins were severely decreased at 20% O_2_ upon FDX2 loss (Figs. 4D and S5*C*). These findings based on proteome analysis were confirmed by immunoassays (Fig. 4*E*).

In mitochondria Fe-S proteins play essential roles in the electron transport chain (ETC) (1, 2). Our data indicates that decreased levels of ETC-associated Fe-S proteins seen in FDX2-deficient cells grown in 20% O_2_ are significantly rescued when cells are grown at 3% O_2_ (Figs. 4*D* and S5*C*). Thus, we focused on effects of FDX2 loss on ETC under low oxygen conditions. Western blot analysis revealed that in cells grown at 20% O_2_, FDX2 loss (promoted by Doxy withdrawal) significantly decreased protein levels of NDUFB8 and SDHB, which are respective subunits of complex I and II, relative to + Doxy controls, an outcome partially but reproducibly rescued at 3% O_2_ (Fig. S5*C*). Consistently, the mitochondrial oxygen consumption rate (OCR) in FDX2-deficient and -proficient ES2 cells grown at 3% O_2_ was comparable (Fig. 4*F*). Furthermore, JC-10 assays of mitochondrial membrane potential showed comparable FCCP-sensitive depolarization in both FDX2-proficient and -deficient groups (Fig. 4*G*). These results indicate that FDX2-deficient cells can maintain ETC function at 3% O_2_, which may, at least in part, explain why FDX2-deficient ES2 cells are able to proliferate under low oxygen conditions.

Finally, we asked whether FDX1, an FDX2 paralog, contributes to FDX2-independent proliferation of ES2 cells under low oxygen. To do so, we knocked out FDX1 by genome editing in FDX2-iKO ES2 cells (Fig. S6*A*). FDX1 knockout did not affect proliferation of FDX2-proficient cells (*i.e.*, +Doxy) either at 20% or 3% oxygen (Fig. S6*B*, left). In FDX2-deficient (-Doxy) conditions, FDX1 knockout slightly increased, rather than suppressed, cell proliferation (Fig. S6*B*, right), arguing against a compensatory role for FDX1 in FDX2 loss.

## Discussion

In this study, we show that FDX2 is essential for tumor initiation and metastasis but not for growth of established tumors in mouse transplantation models based on ES2 ovarian cancer cells (Fig. 2). These findings suggest that the demand for *de novo* Fe-S cluster biosynthesis may be higher during tumor initiation and metastatic stages than it is during tumor growth (Fig. 2). Furthermore, we report that under low oxygen conditions, ES2 cells can proliferate without FDX2, recapitulating the FDX2-independent growth of tumors in subcutaneous and peritoneal dissemination models. We conclude that low oxygen mitigates the downregulation of Fe-S proteins induced by FDX2 loss (Figs. 3 and 4).

Previous studies of the relationship between oxygen and Fe-S cluster biology have focused primarily on functions of Fe-S proteins under hyperoxia (12, 13). For example, NFS1 expressed in tumor cells reportedly exerts anti-ferroptotic activity in high oxygen environments (13). Baik *et al.* more broadly demonstrated that hyperoxia induces a global reduction in levels of Fe-S proteins (12). In contrast, we show that low oxygen markedly attenuated cytotoxicity promoted by FDX2 deficiency (Fig. 3). Accordingly, we observed that low oxygen increased steady-state levels of Fe-S proteins, suggesting a novel paradigm of how cells adapt to reduced oxygen availability (Fig. 4). Proteomic analyses of FDX2-deficient cells under normoxic and hypoxic conditions have been previously reported (24). However, Joshi *et al.* (24) primarily focused on FDX1-dependent protein lipoylation and reported that defects in protein lipoylation caused by FDX1 deficiency were not rescued under hypoxic conditions (1% O_2_). In contrast, our study identified increased Fe–S protein levels under low oxygen conditions, a phenomenon not reported by Joshi *et al.* Hypoxic responses are most commonly associated with regulation by HIF family transcription factors (36); however, HIF activation under hypoxia is transient, and mechanisms underlying chronic hypoxic adaptation remain poorly understood, with only a few exceptions, such as the PLP–PNPO system (37). Here, we demonstrate that prolonged exposure to low oxygen increases levels of Fe-S proteins, raising the possibility that Fe-S protein stabilization under hypoxia may help cells adapt to low oxygen.

Here, we primarily focused on the impact of FDX2 deficiency on the ETC; however, under low oxygen conditions, levels of Fe-S proteins functioning in cellular processes such as DNA repair also appeared to be maintained, even in the absence of FDX2 (Fig. 3). Our findings also raise an important question relevant to how Fe-S cluster biosynthesis proceeds in FDX2-deficient cells. Although low oxygen may protect Fe-S clusters and Fe-S proteins from oxidative stress, the ability of FDX2-deficient cells to sustain long-term proliferation, tumor growth, and ETC function suggests that a low level of Fe-S cluster biosynthesis persists in the absence of FDX2. In this context, Ast *et al.* proposed that Fe-S cluster biosynthesis under hypoxic conditions can proceed independently of the FXN subunit of the Fe-S cluster assembly core complex (19). A similar bypass mechanism might also exist for FDX2, although such FDX2-independent Fe-S cluster biosynthesis requires further experimental confirmation. However, *in vitro* reconstitution experiments showed that FXN-independent Fe-S cluster biosynthesis is substantially reduced relative to FXN-dependent activity (by approximately one-third) (19). These observations are consistent with a model in which such bypass activity is limited but may become sufficient under conditions where demand for *de novo* Fe-S cluster biosynthesis is reduced.

Previous studies have noted a correlation between tumor-initiating activity and metastatic capacity of tumor cells (29, 30). In metastatic colonization, tumor cells must withstand various environmental stresses, such as metabolic stress. For example, tumor cells must rapidly adapt to extremely low oxygen and nutrient conditions before the establishment of vascular support. In these microenvironments, Fe-S proteins functioning in mitochondrial respiration, DNA replication/repair, and other cellular functions may constitute critical bottlenecks, and their dysfunction seen followiong FDX2 loss could robustly impair tumor initiating activity (Fig. 2, *A* and *G*). Anti-ferroptotic FDX2 activity might be a candidate mechanism, since FDX2-deficient cells exhibit increased sensitivity to ferroptosis even under low oxygen (Fig. 3). In striking contrast, we observed that growth of established tumors was virtually unaffected by FDX2 deficiency (Fig. 2*B*), suggesting that once a tumor is established, metabolic redundancy and adaptation to chronic hypoxia may render it more resistant to changes in Fe-S cluster biosynthesis. Our iKO system, which allows inducible FDX2 deletion, should be a valuable tool to further characterise these mechanisms.

In summary, our study reveals that FDX2 is essential for tumor initiation and metastasis but dispensable for growth of established tumors, indicating stage-specific demands for Fe-S cluster biosynthesis: during tumor initiation, putative non-canonical FDX2-independent Fe-S cluster synthesis, if any, may be insufficient to meet these demands. These findings suggest that a requirement for Fe-S clusters during tumor initiation and metastasis could represent a specific cancer cell vulnerability, with potential implications for therapeutic strategies. We further show that low oxygen counteracts cytotoxicity after FDX2 loss and helps stabilize Fe-S proteins, illustrating a potentially novel adaptation to chronic hypoxia.

### Limitations of this study

While we confirmed phenotypes associated with FDX2 deficiency *in vitro* in multiple ES2 clones and also observed FDX2-independent proliferation under low oxygen conditions in JHOC5 cells, we performed transplantation experiments using a single clone or its derivatives. Therefore, we cannot exclude the possibility that clonal variation or cellular background may influence phenotypes observed *in vivo*.

## Experimental procedures

### Reagents

Fluorescent probes FerroOrange and JC-10 were purchased from Dojindo. BODIPY-C11 was purchased from Thermo Fisher Scientific. ML-162 was obtained from Cayman. iFSP1 and Ferrostatin-1 was purchased from MedChemExpress. Doxycycline (Doxy) was purchased from TAKARA BIO. VivoGlo luciferin was purchased from Promega.

### Cell culture

The human OVC lines JHOC5 and ES2 were obtained from Riken Bioresource Center and ATCC, respectively, and maintained in high-glucose DMEM medium supplemented with 10% FCS. 293T cells were cultured in RPMI1640 with 10% FCS. All lines were verified as mycoplasma-free using a MycoAlert kit (Lonza). Cell line authentication tests were not performed. All cells were cultured under 5% CO_2_ at 37 °C. For low oxygen experiments, O_2_ concentrations were set to 3%.

### Lentiviral gene expression and knockout

Lentivirus was produced in 293T cells using standard procedures with FuGENE HD (Promega), psPAX2 and pMD2.G packaging plasmids (CELLECTA), and lentivirus plasmids, as described (38). Lentivirus plasmids pLV-TRE-HA/FDX2 (GER)-Neo, LV-tTS/rtTA-Hyg, LV-Cas9/Bsd-hFDX1 sgRNA #1, LV-Cas9/Bsd-hFDX1 sgRNA #2, LV-Cas9/Bsd-Scramble sgRNA #1, and LV-Cas9/Bsd-Scramble sgRNA #2 were sourced from Vector Builder (Chicago, IL). The sgRNA sequences were as follows: FDX1 sgRNA #1, 5′-TTCTGCTGTCCTCGGCGGCC-3′; FDX1 sgRNA #2, 5′-GTCGCTGAGCGTG-TCGGCGC-3′; scramble sgRNA #1, 5′-GTGTAGTTCGACCATTCGTG-3′; scramble sgRNA #2, 5′-GTTCAGGATCACGTTACCGC-3′. Unless specified, virus infections were performed using a 1:1-2 mixture of 293T culture supernatant and fresh medium. Polybrene was added at 8 ug/ml to the virus mixture to promote infection. Cells transduced with the Cas9/sgRNA construct were selected in 10 ug/ml blasticidin-S (Merk) and analyzed in bulk.

### Cell number determinations

Cell proliferation was continuously monitored using the IncuCyte cell analyzer (Sartorius). Alternatively, relative cell numbers at endpoints were determined by sulforhodamine B staining, as described (39).

### Establishment of FDX2 inducible-KO (iKO) cells

ES2 cells were transduced with tTS and rtTA using a lentivirus vector to produce ES2-TetOn cells. Cells were selected in 250 μg/ml Hygromycin and transduced with a gene cassette expressing FDX2 (GER) (for Genome-Editing Resistant)-HA cDNA driven by the TRE promotor using a lentivirus vector. FDX2 (GER) carries silent mutations at the FDX2_CC2 sgRNA target site. Resulting ES2-TetON-FDX2 (GER)/HA cells were selected in 1000 μg/ml Geneticin. To delete the endogenous gene, we transfected ES2-TetON-FDX2 (GER)-HA cells grown in the presence of 100 ng/ml Doxy with RNP complexes consisting of Cas9 protein and FDX2_CC2 sgRNA using NEPA gene electroporator (37) (Nepa Gene) at the following settings: poring-pulse was set for 150 V with a 5 msec pulse-width, and the transfer-pulse was set for 20 V with a 50 msec pulse-width. RNP-transfected cells were subjected to a cloning procedure using the standard limiting dilution method in medium supplemented with 100 ng/ml Doxy. sgRNA FDX2_CC2 was designed using the ChopChop program (https://chopchop.cbu.uib.no/) and synthesized by GenScript, as described previously (24). The FDX2_CC2 sequence is as follows; 5′-mU∗mC∗mG∗rUrArGrArCrCrGrCrUrCrArGrGrCrCrArGrGrUrUrUrUrArGrArGrCrUrArGrArArArUrArGrCrArArGrUrUrArArArArUrArArGrGrCrUrArGrUrCrCrGrUrUrArUrCrArArCrUrUrGrArArArArArGrUrGrGrCrArCrCrGrArGrUrCrGrGrUrGrCrU∗mU∗mU∗mU-3′ (m and ∗ denote 2′ O-Methyl RNA and phosphorothioate, respectively). KO clones were identified by Western blotting. Two FDX2-iKO ES2 clones were established, and their phenotypic similarities were confirmed. After establishment, clones were cultured in 30 to 100 ng/ml Doxy. Unless stated, we show results obtained with clone #1. Establishment of a FDX2-iKO JHOC5 clone is described elsewhere (25). To induce FDX2 deficiency, cells were pre-cultured 4 days with or without Dox and seeded into new dishes or plates. A real-time cell proliferation assay was performed using IncuCyte.

### Western blot analysis and capillary-based immunoassays

Cells were lysed by sonication using a BIORUPTOR device (SonicBio Co) in RIPA buffer supplemented with protease inhibitors. Lysate protein concentrations were determined using a DC assay kit (Bio-Rad). SDS-PAGE was performed using 4 to 20% Mini-PROTEAN TGX Precast Protein Gels. Proteins were transferred to PVDF membranes using a Transblot Turbo blotting system (BioRad). After incubation with the indicated antibodies, signals were visualized using Fusion Solo imaging system (Vilber, Châtenay-Malabry). Alternatively, protein lysates were analyzed using a capillary-based immunoassay system (JESS Simple Western system, ProteinSimple). Antibodies used were anti-FDX2 antiserum (19), anti-SDHB, anti-ERCC2, and anti-FDX1 (ProteinTech), anti-p53, anti-CHK1, anti-pS345-CHK1, anti-CHK2, anti-pT68-CHK2, anti-DNA-PKcs, and anti-pS2056-DNA-PKcs (CST), anti-Actin (Merck), anti-HSP60 (Protein Simple), and anti-Tubulin-alpha (MBL). Second antibodies used were anti-mouse IgG-HRP and anti-rabbit IgG-HRP for Western blotting. Anti-mouse Ig-HRP and anti-rabbit Ig-HRP (all purchased from Protein Simple) were used for the JESS system. Antibodies were validated using various assays, including Western blotting/JESS analysis.

### EdU-incorporation assay

Cells were cultured in 24 well-plates, and pulse-labeled with 5 μM EdU for 30 min. After fixation in 3.7% formaldehyde/PBS for 10 min, EdU incorporation was detected using a Click-iT Alexa-488 EdU-imaging kit (Thermo Fisher Scientific) according to the manufacturer’s instructions. Fluorescent images were obtained using a BZ-X800 microscope (KEYENCE). The percentages of EdU-positive cells were determined using Hybrid Cell Count software (KEYENCE), and at least 1000 cells were counted for each biological replicate.

### Measurement of cellular free Fe^2+^

FDX2-iKO cells precultured 4 days with or without doxycycline were seeded into 96-well plates. On the next day, cells were stained with FerroOrange. Cells on replicate plates were formaldehyde-fixed and DAPI-stained for normalization. All fluorescent signals were measured using a Synergy H1 plate reader (BioTek, Winooski, VT).

### RNA-seq and data analysis

Total RNAs were prepared from FDX2-iKO ES2 cells cultured 6 days with or without Doxy at 20% O_2_. Alternatively, cells were cultured for an additional 3 days at 3% O_2_ after a 4-day preculture at 20% O_2_. mRNA was purified from total RNA using poly-T oligo-attached magnetic beads. After fragmentation, the first strand cDNA was synthesized using random hexamer primers, followed by the second strand cDNA synthesis. The library was ready after end repair, A-tailing, adapter ligation, size selection, amplification, and purification. 5′-ends of each library were phosphorylated and cyclized. Subsequently, loop amplification was performed to generate DNA nanoballs. Libraries were sequenced on the DNBSEQ-T7 platform (MGI Tech, Shenzhen, China) with paired-end 150 bp reads. GSEA was performed using the RNAseqChef platform (39) (https://imeg-ku.shinyapps.io/RNAseqChef/). Hierarchical clustering analysis and heatmap visualization (Fig. S4*A*) were performed in R (v4.3.3) using normalized counts generated by DESeq2 (v1.42.1). Genes included in the MSigDB gene set “SEMENZA_HIF1_TARGETS. v2025.1.HsHIF-1” were defined as HIF-1 target genes.

### Proteome analysis

Protein samples were prepared from FDX2-iKO ES2 cells cultured 6 days with or without Doxy at 20% O_2_. Alternatively, cells were cultured for an additional 3 days at 3% O_2_ after a 4-days of preculture at 20% O_2_. Proteome analysis based on the LC-MS/MS method was performed using the services of Kazusa DNA Research Institute (Kisarazu, Japan).

Cell lysates were prepared in 100 mM Tris-HCl (pH 8.0), 4% SDS, 20 mM NaCl, and 10% acetonitrile, followed by sonication. No protease inhibitors were used. Protein concentration was determined using a ProteoAnalyzer (Agilent, Santa Clara, CA) and adjusted to 75 ng/μl. Proteins were processed with the SP3 bead method (Cytiva, Washington, D.C) and digested overnight at 37 °C with Trypsin/Lys-C mix (Promega). No variable modifications were included in the database search. After reduction/alkylation and desalting with GL-Tip SDB (GL Sciences, Tokyo, Japan), peptides were quantified by fluorometric peptide assay (Thermo Fisher Scientific), dried, and reconstituted in 0.1% TFA. Peptide mixtures (200 ng, technical replicate n = 1) were analyzed on a Vanquish Neo LC system (Thermo Fisher Scientific) coupled to an Orbitrap Exploris 480 mass spectrometer (Thermo Fisher Scientific). Peptides were separated on a 75 μm × 300 mm C18 column (1.7 μm particle size, CoAnn Technologies) with a linear gradient of solvent A (0.1% formic acid in water) and solvent B (0.1% formic acid in 80% acetonitrile) at a flow rate of 250 nl/min, with the column maintained at 50 °C. The Orbitrap was operated in positive ion mode with a spray voltage of 2.0 kV and a capillary temperature of 275 °C. Full MS1 scans were acquired at 30,000 resolution over an m/z range of 495 to 745. Data-independent MS2 scans (30 windows, 8 Th isolation width) were acquired over m/z 200 to 1800 at 15,000 resolution using higher-energy collisional dissociation (HCD) with a normalized collision energy of 27. Automatic gain control (AGC) target values and maximum injection times were set to instrument defaults for DIA mode. Data were analyzed with DIA-NN 2.2.0 Enterprise software. A predicted spectral library was generated using the Human UniProtKB/Swiss-Prot database (Proteome ID UP000005640, release 2025_04). Peptide-spectrum matching included deep learning–based predictions of spectra, retention times, and ion mobilities. Protein identification and quantification were performed with a false discovery rate (FDR) cutoff of 1% at both precursor and protein levels. Quantification used the QuantUMS algorithm with RT-dependent normalization across runs. The mass spectrometry proteomics data have been deposited to the JPOST repository (JPST004079) (40). For peer review, the dataset is accessible at https://repository.jpostdb.org/preview/203586657868ca1eaecb4e1, using the access key 1070. The data will be publicly available upon acceptance of the manuscript.

Data analysis was performed as follows. First, proteins judged to be ND (not detected, or below detection limit) in all four experimental groups (20% O_2_/+ Doxy, 20% O_2_/- Doxy, 3% O_2_/+ Doxy, and 3% O_2_/- Doxy) were excluded from analysis. For the remaining proteins, ND values were imputed by substituting one-half of the minimum corrected expression value observed among non-ND measurements.

### Lipid peroxidation assay

Lipid peroxidation was assessed using a BODIPY-C11 fluorescent probe. After a 4-days preculture with or without Doxy at 20% O_2_, cells were cultured 2 days, treated 2 h with ML-162 at 3% O_2_, and then incubated with 5 μM BODYPI-C11 for 15 more min. Fluorescent BODIPY-C11 signals were detected using a SA3800 flowcytometer (Sony) at FITC detection settings designed to quantify oxidized BODIPY C11, as described previously (25).

### Mouse experiments

Animal experiments were performed with approval of the Miyagi Cancer Center Research Institute Animal Care and Use Committee. Mice were maintained in specific pathogen-free (SPF) facilities with a 12-h light-dark cycle. Unless stated, mice were fed a normal diet (MF from Oriental Yeast Co., LTD) ad libitum.

In *sc* transplantation experiments, nude mice were inoculated in the dorsal flank with FDX2-iKO cells at 1 x 10ˆ6 cells plus Matrigel (50%) per site. In *iv* models, each mouse received 7 × 10ˆ5 cells *via* tail vein injection. In *ip* models, each mouse received 3 × 10ˆ5 or 1 × 10ˆ6 cells per injection and underwent bioluminescence imaging (see below) on day 2. Mice with luminescent signals < 1% of the mean signal of all recipients were considered inoculation failures and excluded from further analyses. After transplantation, mice were immediately shifted from a normal diet to either a Doxy-containing (200 ppm; LabDiet #5TP7) or a Doxy-free (LabDiet #5001) diet, both obtained from PMI Nutrition International.

In *sc* models, tumor length and width were measured by calipers, and volume calculated based on the standard formula: (length x width2)/2. For *in vivo* luciferase imaging, mice were anesthetized with 2 to 2.5% isoflurane and ip-injected with VivoGlo luciferin (100 mg/0.1 ml PBS). Bioluminescent signals were acquired using IVIS Lumina III (PerkinElmer, Waltham, MA) 10 min post-injection under constant anesthesia. Data analyses were done using Living Image v4.5 software (PerkinElmer). Regions of interest (ROIs) were drawn over the upper body, and total photon flux (photons/sec) was quantified.

### Immunohistochemical analyses

Immunohistochemical staining was performed using a Ventana instrument and reagents (Roche). For immunostaining of tissue sections, antigen retrieval was not performed except for staining with the anti-Ki67 antibody (Roche). Anti-HA and anti-CA9 antibodies were obtained from ProteinTech and Abcam, respectively.

### Measurement of oxygen consumption rates

Cells were seeded into XF96 cell culture plates and incubated overnight at 3% O_2_ to allow attachment. OCRs were measured using a Seahorse XF96 flux analyzer (Agilent). The assay medium was DMEM (unbuffered) containing 4500 mg/L glucose, 0.29 mg/ml glutamine and no pyruvate. After measuring basal OCR, rotenone and antimycin A1 were added at 3 μM each to inhibit electron transport. Rotenone/antimycin A-sensitive OCR was considered mitochondrial OCR. After analysis, cells were fixed with trichloroacetic acid and stained with sulphorhodamine B (Sigma). OCR values were normalized to the number of cells, as described previously (41).

### Statistical analysis

No statistical methods were used to predetermine sample size. Experiments were not randomized, nor were investigators blinded to allocation during experiments and outcome assessment. Normality of data distribution was assessed using the Shapiro–Wilk test in GraphPad Prism. Student’s *t* test (2-tailed), the Mann–Whitney *U* test (2-tailed) or a one-way ANOVA followed by Tukey’s *post hoc* test were used when comparing two or multiple groups, respectively. A *p* value of <0.05 was considered significant. Data are presented as mean with the range or SEM.

## Data availability

Proteomics data have been deposited in JPOST (JPST004079). RNA-seq data have been deposited in the Gene Expression Omnibus (GEO) (GSE324929). Both datasets will be publicly available upon publication of this article. Source data are provided with this paper as a Source Data file. All unique materials used in this study are available upon request.

## Supporting information

This article contains supporting information.

## Conflict of interest

The authors declare that they have no conflicts of interest with the contents of this article.
