# Supplementary material for: The iron-sulfur cluster assembly factor FDX2 is required for tumor initiation but not for growth of established tumors in transplantation models
Source: J Biol Chem. 2026 May 27;302(7):113200. doi: 10.1016/j.jbc.2026.113200 (PMC13284478; doi:10.1016/j.jbc.2026.113200)
Supplement: Supplementary Figures [file mmc2.pdf]

Supporting information

The iron-sulfur cluster assembly factor FDX2 is required for tumor initiation but not for growth of established tumors in transplantation models.

Eifumi Hashimoto, Mai Ohuchi, Miyuki Nomura, Shuko Miyahara, Kayoko Hayashi, Masatoshi Saito, Yoji Yamashita, Muneaki Shimada, Hidekazu Yamada, Nobuhiro Tanuma

- Figure S1, related to Fig. 1
- Figure S2, related to Fig. 2
- Figure S3, related to Fig. 2
- Figure S4, related to Fig. 3
- Figure S5, related to Fig. 4
- Figure S6, related to Fig. 4

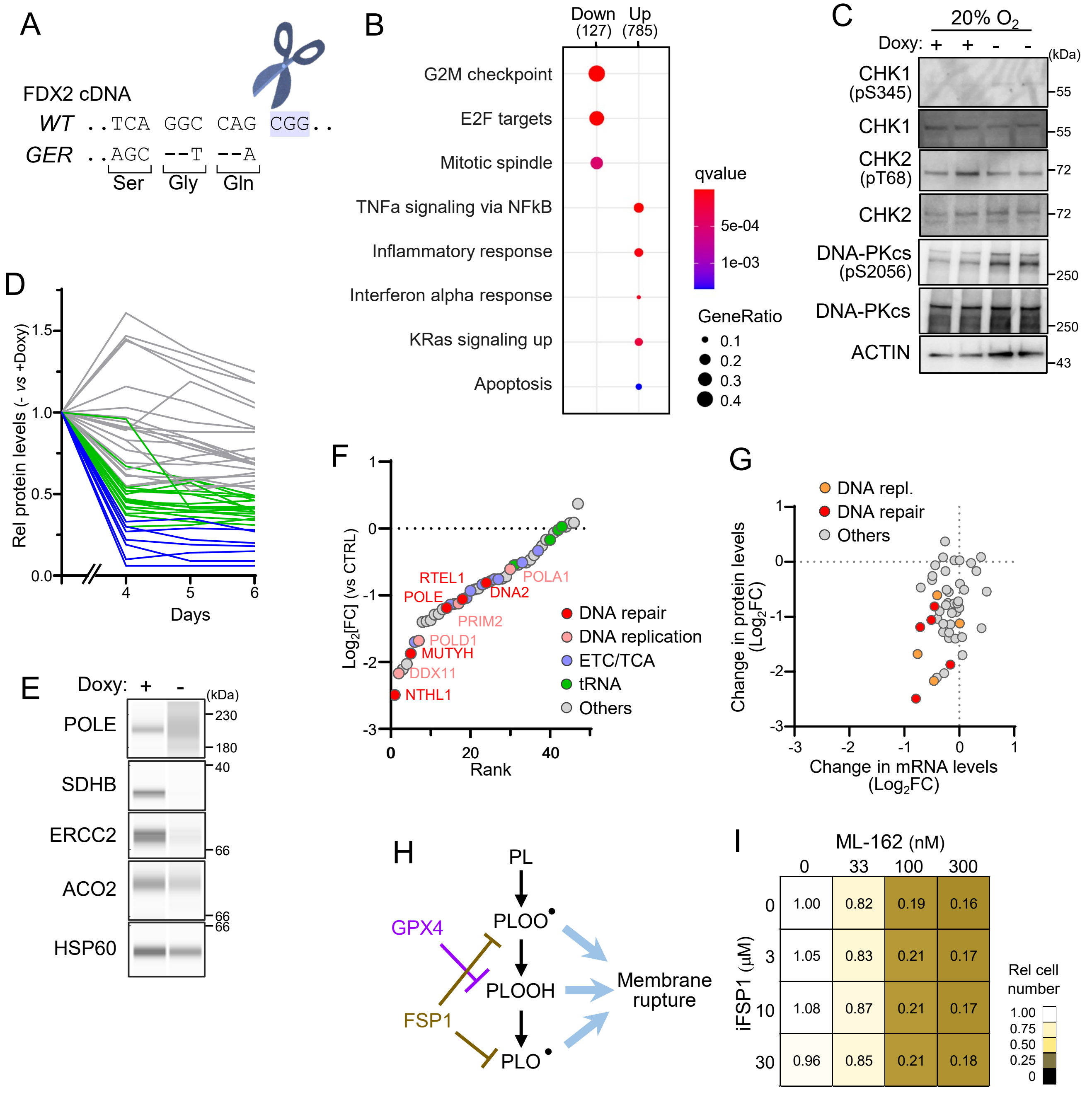

Figure S1. Analysis of FDX2-iKO ES2 cells under ambient oxygen, related to Figure 1.

(A) Shown is a short portion of WT and GER-type human FDX2 cDNA sequences. The GER mutant harbors silent mutations within the FDX2 sgRNA target site. PAM sequence is blue-highlighted.

(B) GSEA analysis of RNA-seq (performed as in Figure 1F) data from FDX2-iKO ES2 cells.

(C) Levels of phosphorylated CHK1, CHK2 and DNA-PKcs in FDX2-iKO ES2 cells analyzed as in Fig 1G. Anti-DNA-PKcs (phosphorylated and total) and anti-ACTIN blots are the same as those in Fig 1G.

(D) Time course of Fe-S protein decrease following FDX2 depletion. FDX2-iKO ES2 cells were cultured with or without Doxy starting at Day 0. For analysis, cells cultured in −Doxy conditions were collected on days 4, 5, and 6, while Control (+Doxy) were collected on day 4. Relative Fe–S protein levels in −Doxy conditions on days 4, 5, and 6 were then normalized to the +Doxy control on day 4 and plotted. Each line represents an individual Fe–S protein. Lines were colored based on relative values obtained on day 6: blue, <0.3; green, 0.3–0.5; and gray, >0.5.

(E) Levels of representative Fe-S proteins in FDX2-iKO cells analyzed by capillary-based immunoassays. HSP60 served as loading control.

(F) Rank plot of all Fe-S proteins detected in proteome analysis. Proteins were ranked based on the degree of change in their levels after FDX2 depletion. Color code indicates function of each protein.

(G) 2D plot showing relationship between FDX2 depletion-induced changes in levels of either proteins or mRNAs encoding Fe-S proteins shown in G. Fe-S proteins related to DNA replication or repair are color-coded as in F.

(H). Scheme showing phospholipid (PL) oxidation associated with cell membrane rupture and subsequent ferroptosis. GPX4 and FSP1 protect cells from ferroptosis by decreasing PLOOH levels and scavenging PL radicals, respectively.

(I) Effects of single or dual GPX4/FSP1 inhibition on FDX2-iKO cell viability. FDX2-iKO cells cultured in Doxy were left either untreated, or treated 1 day with ML-162 and/or iFSP1 at concentrations shown along respective Y- and X-axes. Relative cell number was assessed by SRB staining and reported in boxes as the proportions of viable cells in each treatment group to viable untreated cells (the latter set to 1.0). n = 6 biological replicates.

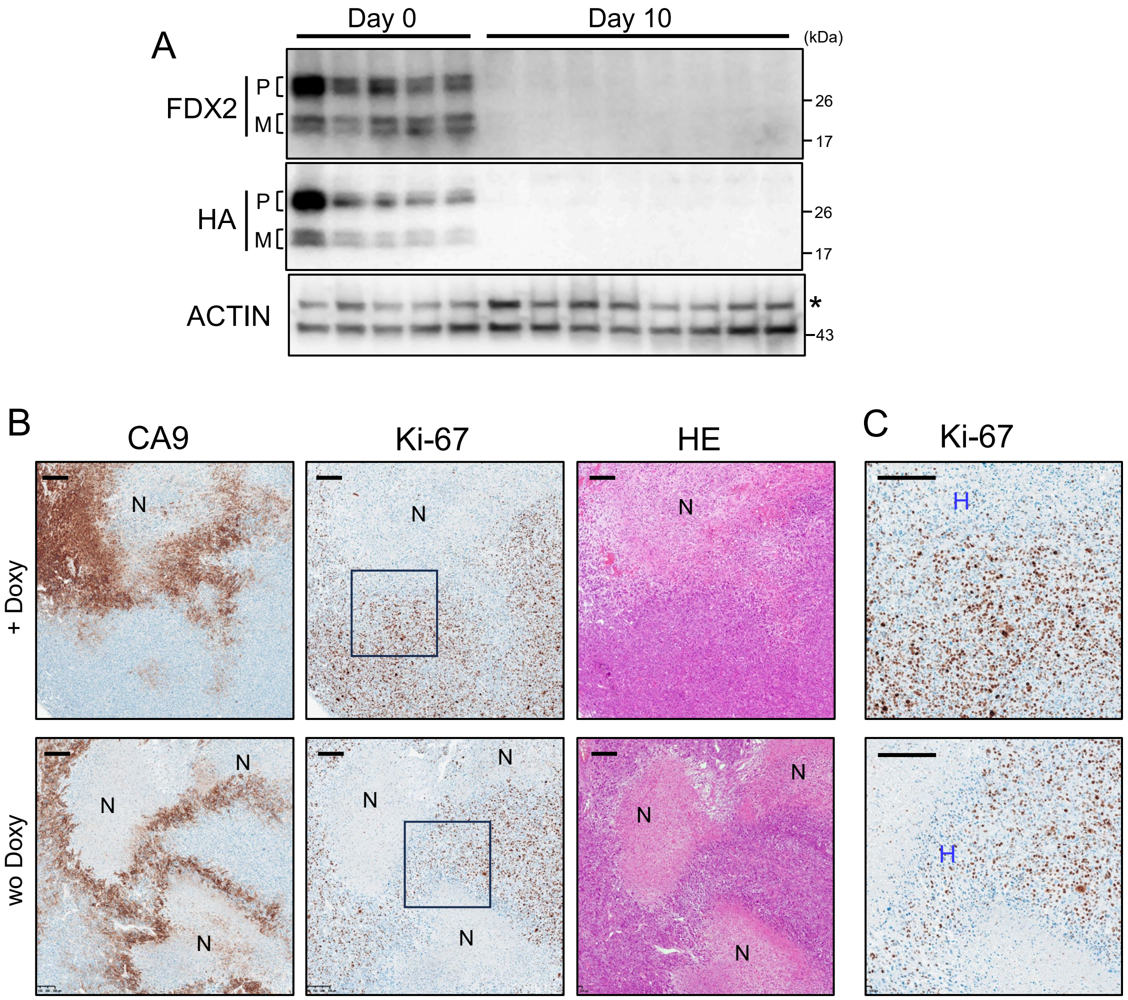

Figure S2. Analysis of FDX2-iKO ES2 tumors in subcutaneous transplantation models, related to Figure 2.

(A) WB analysis of FDX2-iKO tumors from mice that underwent Doxy withdrawal, as described in Fig. 2C. Tumor samples were collected 0 or 10 days after switching mice from a Doxy-containing to Doxy-free diet.

(B) IHC analysis of tumors from mice maintained on Doxy or switched to a Doxy-free diet. Analysis was performed ~2 weeks after diet change. N indicates necrotic areas.

(C) Higher-magnification view of Ki67 staining at boundaries between hypoxic and non-hypoxic regions (boxed in B). H indicates hypoxic areas, as defined by CA9 staining. Scale bars in B and C, 0.2 mm.

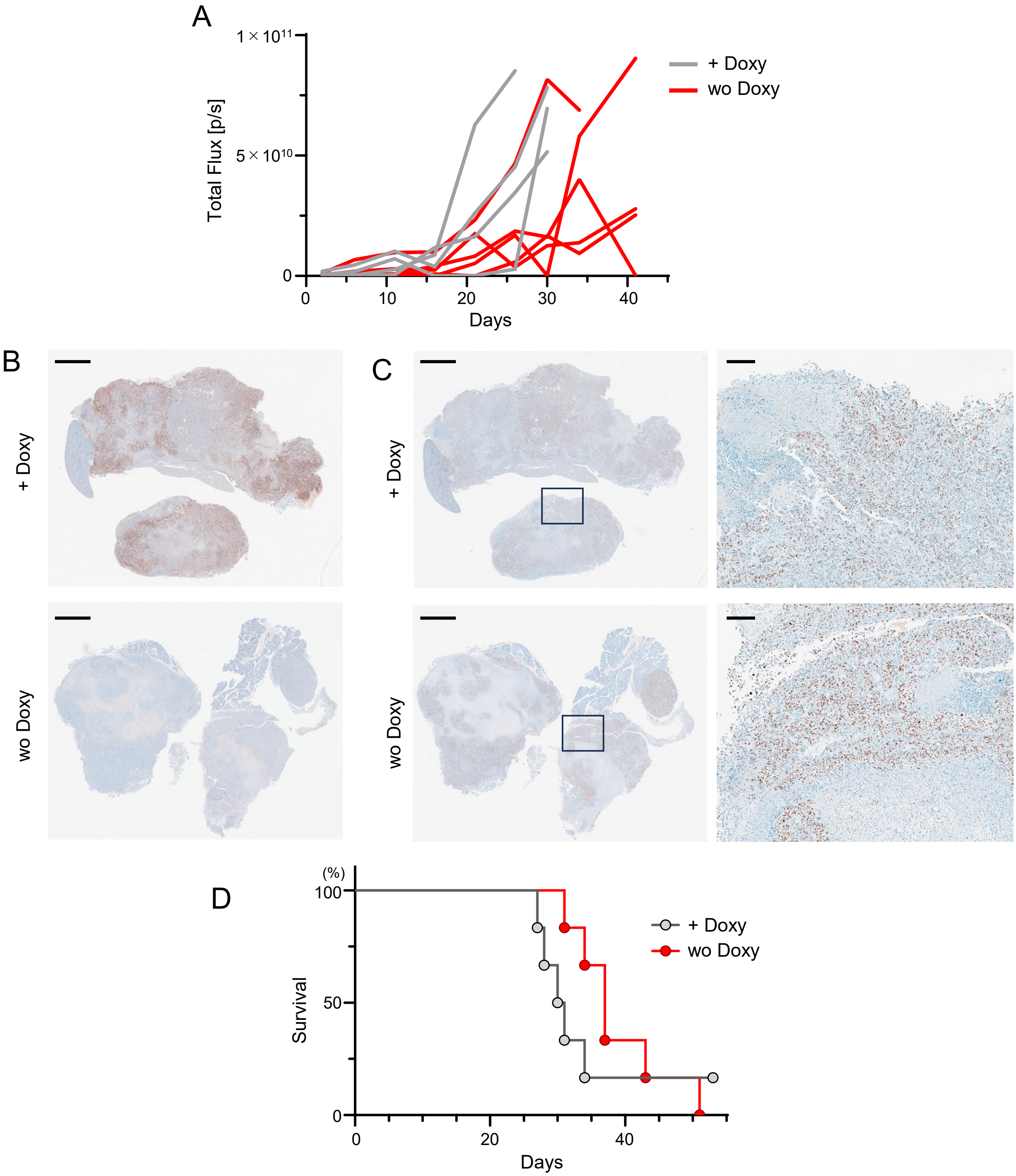

Figure S3. Analysis of FDX2-iKO ES2 tumors in peritoneal dissemination models, related to Figure 2.

(A) Quantification of tumor burden in mice shown in Fig. 2H over time (days 2–41), as monitored by bioluminescent imaging.

(B) IHC analysis of omental metastases from + Doxy and – Doxy groups, stained with an anti- HA antibody to detect FDX2-HA.

(C) Ki-67 staining of serial sections shown in A. Higher-magnification views of boxed areas are shown at right.

(D) Survival of mice implanted ip with a high (1x 106) number of FDX2-iKO cells (grown in vitro with Doxy). Inoculated mice were divided into 2 groups on day 0 and then fed either a Doxy-containing or Doxy-free diet. The number of mice in the with- and without-Doxy groups was n = 6 and n = 8, respectively.

Scale bars, 200 μm (A and B, low magnification) or 20 μm (B, high magnification).

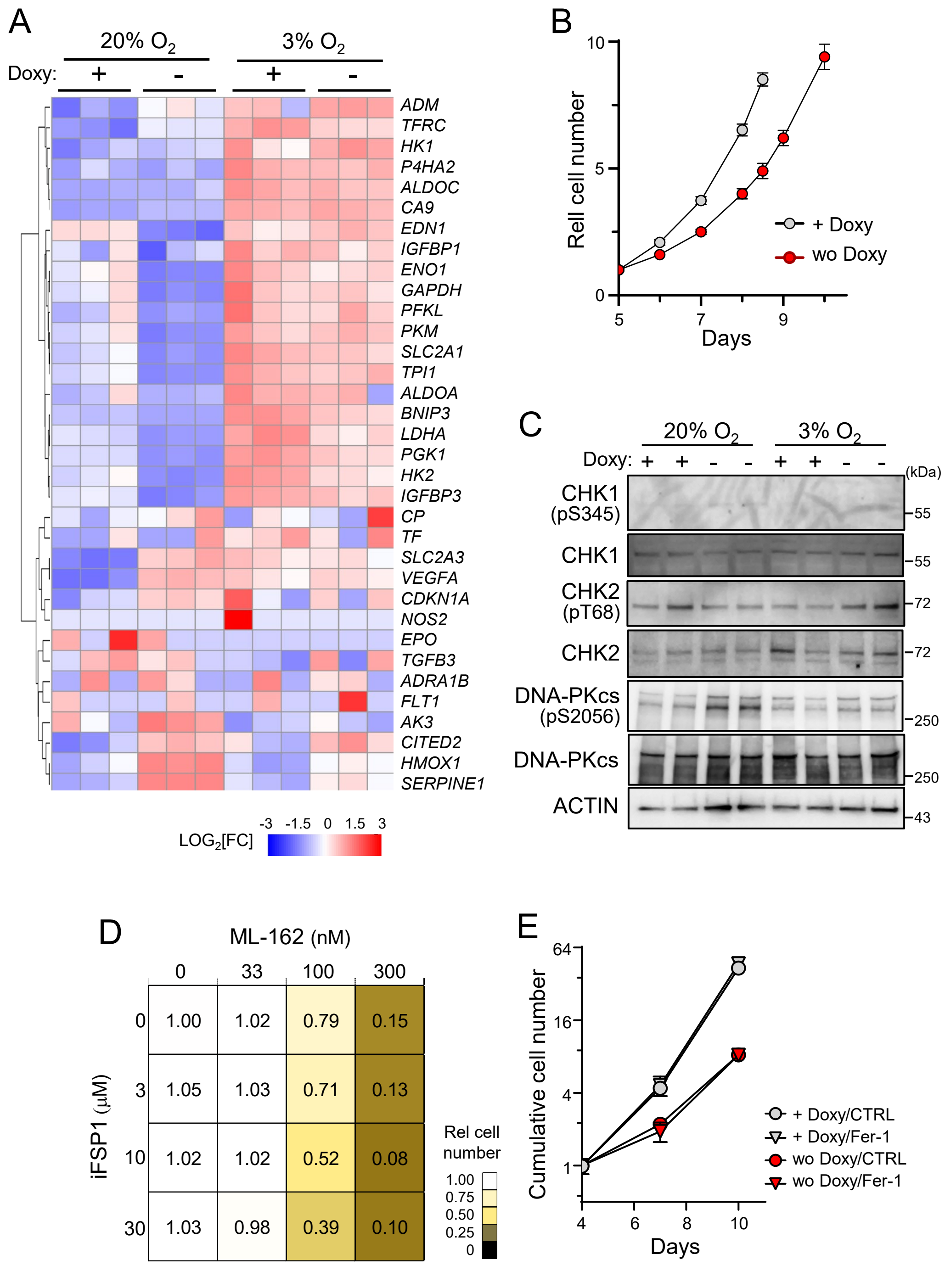

Figure S4. Analysis of FDX2-iKO ES2 cells under low-oxygen conditions, related to Figure 3.

(A) Heatmap of HIF-1 target genes (MSigDB: “HIF1-target genes, Semenza”) in FDX2-iKO cells ± doxy under normoxia or hypoxia (3% O_2) for 6 days. DESeq2-normalized counts are shown as row-wise Z-scores. n = 3 biological replicates.

(B) Proliferation of FDX2-iKO ES2 cells cultured with or without Doxy at 1% O2. n = 3 biological replicates.

(C) Phosphorylation status of CHK1, CHK2 and DNA-PKs before and after induction of FDX2 loss at 3% and 20% O2. Anti-DNA-PKcs (phosphorylated and total) and anti-ACTIN blots are the same as those in Fig. 3D.

(D) Effects of single or dual inhibition of GPX4 and FSP1 at 3% O2, determined as in Fig. S1E. n = 6 biological replicates.

(E) Effects of Ferrostatin-1 on proliferation of FDX2-iKO ES2 cells. Cells were cultured as in Fig. 3B, with or without 10 μM Fer-1.

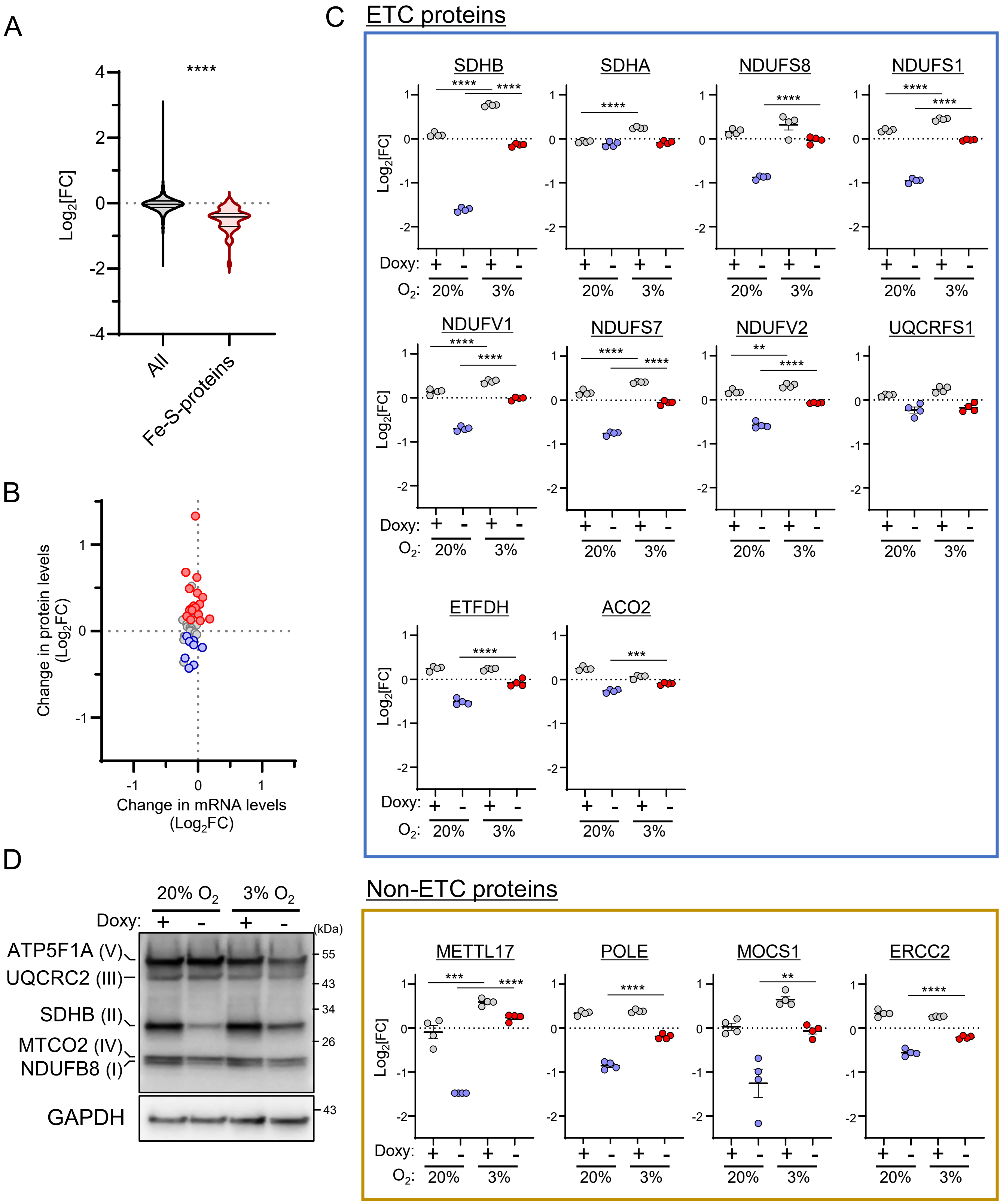

Figure S5. Effects of FDX2-deficiency on levels of ETC and non-ETC in FDX2-iKO ES2 cells grown under ambient or low-oxygen conditions, related to Figure 4.

(A) Reanalysis of Fig. 4A comparing effects of FDX2 knockout at 3% O2 on all proteins versus Fe-S proteins.

(B) 2D plot comparing FDX2 depletion-induced changes in protein and mRNA abundance for the Fe-S proteins shown in Fig. 4B. Symbol colors correspond to those in Fig. 4B.

(C) Shown are relative levels of representative Fe-S proteins associated with ETC (upper) or non-ETC (lower) functions in FDX2-iKO ES2 cells grown in conditions indicated along the x-axes, based on proteome analysis. n = 4 biological replicates.

(D) Western blot analysis of lysates of FDX2-iKO ES2 cells grown in indicated conditions for representative subunits of complexes I–V of the ETC. Complex numbers are indicated in parentheses. Note that SDHB of complex II is an Fe-S protein. GAPDH served as loading control.

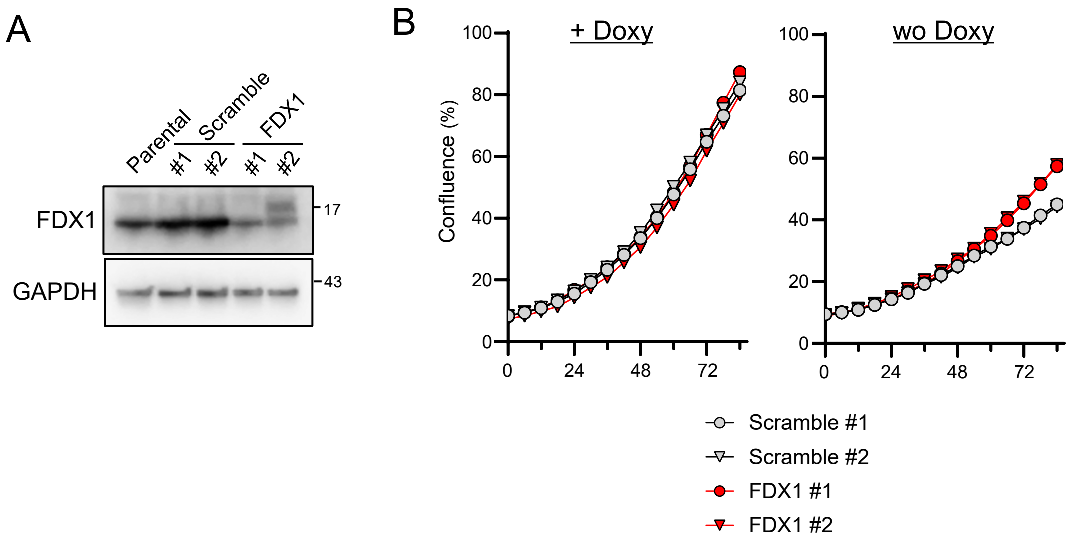

Figure S6. Effects of FDX1 knockout on proliferation of FDX2-proficient and –deficient ES2 cells, related to Figure 4.

(A) Western blot showing FDX1 protein expression in parental FDX2-iKO ES2 cells and in cells transduced with Cas9 and the indicated sgRNA. GAPDH served as loading control.

(B) Proliferation of FDX2-iKO ES2 cells expressing Cas9 and the indicated sgRNA cultured with or without Doxy under 3% O2 conditions. Data are shown as mean ± SEM (n = 3 biological replicates).
